# Supplementary material for: Complexome profiling of the Chlamydomonas psb28 mutant reveals TEF5 as an early PSII assembly factor
Source: Plant Cell. 2025 Mar 17;37(6):koaf055. doi: 10.1093/plcell/koaf055 (PMC12164590; doi:10.1093/plcell/koaf055)
Supplement: koaf055_Supplementary_Data [file koaf055_supplementary_data.zip › psb28_interactive.zip!/plots/intensity/qualityPlots/peptides/psaA_Cre-1.g2717000.t1.p1.html]

### Cre-1.g2717000.t1.p1

Trivial name: psaA  
SeqCoverage [median]: 22.00  
Mass: 83.16 kDa  
  
 

| Sequence | MissedCleavage | Mass [Da] | wtIntensity | muIntensity | Score |
| --- | --- | --- | --- | --- | --- |
| AIMADLYPSFAK | 0 | 1325.67 | 1578729.02 | 2653907.29 | 235.32 |
| ALSITQGR | 0 | 844.48 | 953295.49 | 1155453.69 | 193.86 |
| DYDPTNNYNNLLDR | 0 | 1725.76 | 745907.56 | 943167.13 | 308.60 |
| EILEAHR | 0 | 866.46 | 740431.88 | 577811.60 | 226.65 |
| EIPLPHDLLLNR | 0 | 1428.81 | 1804036.49 | 2850752.18 | 124.12 |
| FSNYEAWLSDPTHIK | 0 | 1806.86 | 31532.89 | 12212.85 | 164.41 |
| GVLFAR | 0 | 661.39 | 0.00 | 3364.03 | 73.97 |
| GYWQELIESIVWAHNK | 0 | 1971.98 | 3610.37 | 0.00 | 118.21 |
| IAVDRNPVETSFEK | 1 | 1603.82 | 2293592.57 | 1794320.74 | 189.07 |
| LIPDKANLGFR | 1 | 1242.71 | 242757.81 | 306338.71 | 117.67 |
| LKVAPAIQPR | 1 | 1091.68 | 8192.08 | 7284.65 | 95.36 |
| LLDAGVDPK | 0 | 926.51 | 2064586.43 | 1600175.40 | 157.91 |
| LLDAGVDPKEIPLPHDLLLNR | 1 | 2337.31 | 20913.41 | 33876.07 | 127.17 |
| NPVETSFEK | 0 | 1049.50 | 305154.62 | 221969.08 | 187.50 |
| PGHFSR | 0 | 699.35 | 15967.93 | 521.23 | 148.53 |
| TISTPER | 0 | 802.42 | 680699.70 | 463851.58 | 157.86 |
| TISTPEREAK | 1 | 1130.59 | 79991.02 | 87799.58 | 229.48 |
| TNWGIGHSMK | 0 | 1129.53 | 164301.76 | 65928.50 | 110.47 |
| TNWGIGHSMKEILEAHR | 1 | 1977.98 | 41223.86 | 9585.82 | 95.65 |
| VAPAIQPR | 0 | 850.50 | 1893033.33 | 1379757.22 | 178.02 |
| VKIAVDRNPVETSFEK | 2 | 1830.98 | 1386.33 | 8277.03 | 51.03 |
| WAKPGHFSR | 1 | 1084.56 | 148067.13 | 68979.03 | 108.43 |
|  |  |  | 13817411.7 | 14245333.4 |  |
